# ﻿Dalodesmid millipedes from alpine and subalpine habitats in New South Wales and Victoria, Australia (Diplopoda, Polydesmida, Dalodesmidae)

**DOI:** 10.3897/zookeys.1262.176273

**Published:** 2025-12-10

**Authors:** Robert Mesibov

**Affiliations:** 1 Ulverstone, Tasmania, Australia Unaffiliated Ulverstone Australia

**Keywords:** Litter sampling, locality records, new genus, new species, pitfall trapping, taxonomy

## Abstract

Eleven new species of Dalodesmidae (Diplopoda, Polydesmida) are described: *Araneopedis porchi***gen. nov. et sp. nov.** (type species), *A. bogong***sp. nov.**, *A. buffalo***sp. nov.**, *A. dargo***sp. nov.**, *A. gibbae***sp. nov.**; *Cernethia dysmica***sp. nov.**; *Dibologonus sladei***gen. nov. et sp. nov.** (type species), *D. major***sp. nov.**, *D. minor***sp. nov.**, *D. oedipus***sp. nov.**; *Polydactylogonus sanctogwinear***gen. nov. et sp. nov.** (type and only species). Males of *D. oedipus***sp. nov.** have modified legpairs 13, 15 or 17, with reduced prefemur, femur and postfemur, and fused, swollen tibia and tarsus. New locality records are presented for the dalodesmids *Cernethia inopinata* Mesibov, 2015, *Lissodesmus milledgei* Mesibov, 2006 and *Orthorhachis durabilis* Mesibov, 2008.

## ﻿Introduction

Only three species of Dalodesmidae have previously been recorded at high-elevation sites in the mountain ranges of eastern Victoria (Vic) and southeastern New South Wales (NSW) (Mesibov 2008a): *Orthorhachis catherinae* Mesibov, 2008 (Kosciusko National Park, NSW, 1320 m), *O. durabilis* Mesibov, 2008 (Mt Buller, Vic, 1560 m) and *O. vinnula* Mesibov, 2008 (Bennison Plains, Vic, 1310 m). In this paper I describe 11 new alpine and subalpine dalodesmid millipedes from an invertebrate survey conducted in the Australian Alps in 2023–25 by Nicholas Porch (Deakin University) and his students. The survey also returned new localities, reported here, for three previously described dalodesmids. A twelfth new species from the survey, *Procophorella bawbawensis* Mesibov, 2025, has been separately described and its genus newly placed in Dalodesmidae (Mesibov 2025).

## ﻿Materials and methods

All specimens studied came either from litter samples (2023, 2024, 2025) or pitfall traps (2024). Leaf litter samples were collected and Tullgren funnel-extracted by Nicholas Porch and his students. Specimen lots dated 2023 were from single 2 m^2^ litter patches, and 2024 and 2025 lots were from four combined 0.25 m^2^ patches collected within 10 m of a pitfall grid. In the type details below, the two methods are referred to as “2 m^2^ litter sample” and “1 m^2^ litter sample”, respectively. Pitfall grids at each site comprised 16 plastic tubes set in a 4 × 4 square with traps ca 2–3 m apart. Traps were filled with 25 ml of 80% propylene glycol and removed after approximately two weeks. All specimens of the new species are preserved in 70% or 95% ethanol in Museums Victoria.

Selected specimens were dissected for scanning electron microscopy and digital imaging. Whole-animal images were captured either with an OM Systems TG-7 digital camera or with an Omax A35180U3 eyepiece video camera. Gonopods were cleared in 90+% lactic acid, temporarily mounted in a 1:1 glycerol:water mixture and imaged with the Omax camera mounted on an AmScope B210 microscope, with subsequent focus-stacking of manually focused images using Zerene Stacker 1.04 software. Some gonopod figures show the focus-stacked images, while Figs 8B, 14D have outline drawings digitally traced from focus-stacked images. For scanning electron microscopy, material was air-dried and temporarily fixed to stubs prior to platinum coating to 5 nm with a Cressington 208 HR sputter coater and imaging with a Hitachi SU-70 operated in high-vacuum mode.

Maps were generated with QGIS 3.22 (https://qgis.org/) and figures were composed and edited with GIMP 2.10 (https://www.gimp.org/). The base map in all map figures is from OpenTopoMap (https://opentopomap.org), with an overlay of major roads from Data Vic (https://discover.data.vic.gov.au/dataset/vicmap-transport-road-line). All elevations reported are above sea level.

Details of all specimen lots for named Dalodesmidae from the Hermon Slade Foundation project are provided in Suppl. material 1 in Darwin Core format.

In this paper I use the term “solenomere” for a major process on the gonopod telopodite that carries the opening of the prostatic groove. The term is one of convenience for describing and labelling the process, and I am not suggesting that the structure labelled in this way is homologous with similar processes on the telopodites of other Polydesmida.

### ﻿Abbreviations

**ACT** Australian Capital Territory, Australia;

**NSW** New South Wales, Australia;

**VIC** Victoria, Australia;

**NMV** Museums Victoria, Melbourne;

**M** adult male;

**F** adult female;

**J** juvenile.

## ﻿Results

### ﻿Taxonomy

**Order Polydesmida Pocock, 1887**

**Suborder Dalodesmidea Hoffman, 1980**

**Family Dalodesmidae Cook, 1896**

#### ﻿ Araneopedis gen. nov.

Taxon classificationAnimaliaPolydesmidaDalodesmidae

A91EEB5F-18D5-5952-B8AE-CC6DABE5BBD2

https://zoobank.org/83711322-8551-445B-8BD5-B5956B3FA3DA

##### Type species.

*Araneopedis porchi* sp. nov., by present designation.

##### Other assigned species.

*Araneopedis bogong* sp. nov., *A. buffalo* sp. nov., *A. dargo* sp. nov., *A. gibbae* sp. nov.

##### Diagnosis.

Gonopod telopodite split at 1/3 to 1/2 height into large, erect, similarly sized, lateral and medial branches, with a smaller, shorter, separate, posteromedial solenomere. The only other Australasian dalodesmid with a gonopod split into similarly sized branches with a separate solenomere is *Paurodesmus sjoestedti* (Verhoeff, 1924) (Mesibov 2008b), but in that species the solenomere is nearly as large as the medial and lateral branches and arises basally, rather than at ca 1/2 telopodite height.

##### Description.

Small dalodesmids ca 7–9 mm long with body plan head + 19 rings (including telson). Male colour in alcohol pale brownish-yellow, darker on head, distally on antennae and marginally on tergites and metatergites. Head with vertex sparsely setose, frons and clypeus setose; vertigial sulcus reaching ca 1/2 way to level of line between top of antennal sockets. Sockets separated by ca 2× socket diameter. Antenna almost reaching tergite 3 when manipulated. Relative antennomere lengths (6,3) > 2 > (4,5), 6 widest. Relative tergite widths 6 > 5 > 4 > 3 > 2 > collum; rings 6–16 subequal in width, 17 and 18 narrower. Collum much narrower than head, ovoid in dorsal view, corner rounded. Paranotal margin on ring 2 lower than ring 3 margin; large pit ventrally under ring 2 paranotum. Midbody paranotal margin at ca 2/3 ring height, level; paranotum with anterior corner gently convex, laterally very slightly convex and slightly wider behind, with a few short marginal setae laterally and posteriorly; posterior margin more or less straight; posterior corner slightly projecting on last few rings. Metatergites with 3 transverse rows of low bumps carrying short setae where not abraded; anterior row of bumps narrowest, posterior row widest. Limbus an irregular row of blunt doubled spines. Pore formula normal; ozopore opening dorsally at posterolateral corner of paranotum. Epiproct extending a little beyond anal valves, sides slightly concave, apex truncate; spinnerets in square array. Hypoproct trapezoidal. Sternites slightly wider than long, impressions shallow, transverse impression a little deeper than longitudinal impression. Spiracles small, round or slightly ovoid, on diplosegments with posterior spiracle between leg bases. Length of midbody leg ca 1.5–1.7× midbody ring diameter. Relative podomere lengths tarsus >> (prefemur, femur) >> (postfemur, tibia); tarsus very slightly curved. Anterior legs somewhat swollen, prefemur and femur arched dorsally. Numerous sphaerotrichomes on anterior leg tarsus, tibia; sphaerotrichomes with pointed shafts strongly declined and with flattened globular bases. Brush setae on prefemur and femur, tapering to blunt points, often curving proximally. Gonopore opening distomedially on leg 2 coxa. Aperture transversely ovoid, ca 1/2 width of ring 7 prozonite, rim projecting a little posterolaterally. Retracted gonopod telopodites reaching just past leg 6. Ring 6 sternite excavate with short setal brushes laterally, supporting retracted telopodites. Telopodites erect, closely appressed, divided into lateral branch, medial branch and posteromedial solenomere.

Female closely similar to male, with shorter legs; epigynum slightly raised, subrectangular; cyphopods not examined in any species.

##### Name.

Latin *aranea* + *pedis*, “spider-foot”. The well-separated lateral and medial branches on the gonopod telopodite together with the smaller posteromedial solenomere remind me of the two principal claws and smaller median claw on the tarsus of a web-building spider. Gender masculine.

##### Remarks.

The five known species of *Araneopedis* gen. nov. are closely similar in overall appearance and are geographically clustered in the Victorian mountains, with a known genus range of ca 3500 km^2^ (Fig. 15A).

#### ﻿ Araneopedis porchi sp. nov.

Taxon classificationAnimaliaPolydesmidaDalodesmidae

58DE5493-9B3E-5C37-9873-D08DC7FFCA1B

https://zoobank.org/36364101-843C-4060-8689-F17530CE55DE

Figs 1, 2, 15A

##### Type material.

***Holotype*.** Male, Bogong High Plains, Pretty Valley Road, 1.2 km SE of Mt McKay, site HS01-C-L1, -36.8823, 147.2534 ± 25 m, 1725–1745 m, coll. Nicholas Porch, 2024-04-22, 1 m^2^ litter sample from closed shrubby snow gum woodland, NMV K16475. ***Paratypes*.** 6M, 11F, details as for holotype, NMV K16476; 1M, same details but site HS01-C-P1, pitfall sample, NMV K16477; 8M, 11F, same details but 1.25 km SE of Mt McKay, site HS01-C-L2, -36.8824, 147.2537 ± 25 m, 1730–1750 m, 1 m^2^ litter sample, NMV K16465; 1M in 95% EtOH, same details, NMV K16466; 5M, 5F, same details but 1.3 km SE of Mt McKay, site HS01-C-L3, -36.8828, 147.2539 ± 25 m, 1735–1755 m, NMV K16474.

##### Additional material.

51M, 64F, 1J from nine other sites. See Suppl. material 1 for details.

##### Diagnosis.

Adult males distinguished from *Araneopedis buffalo* sp. nov. by the similar sizes of posterior rings vs enlarged rings; from *A. buffalo* sp. nov. and *A. gibbae* sp. nov. by the undivided vs divided lateral branch; from *A. dargo* sp. nov. by the equally tall lateral and medial branches vs much taller medial branch; and from *A. bogong* sp. nov. by the simply curved solenomere vs solenomere with a wide distal-facing concavity.

##### Description.

As for the genus, with the following details. Male/female length ca 7/7 mm, maximum midbody width 0.6/0.6 mm, midbody metatergite width 1.5× prozonite width.

**Figure 1.**
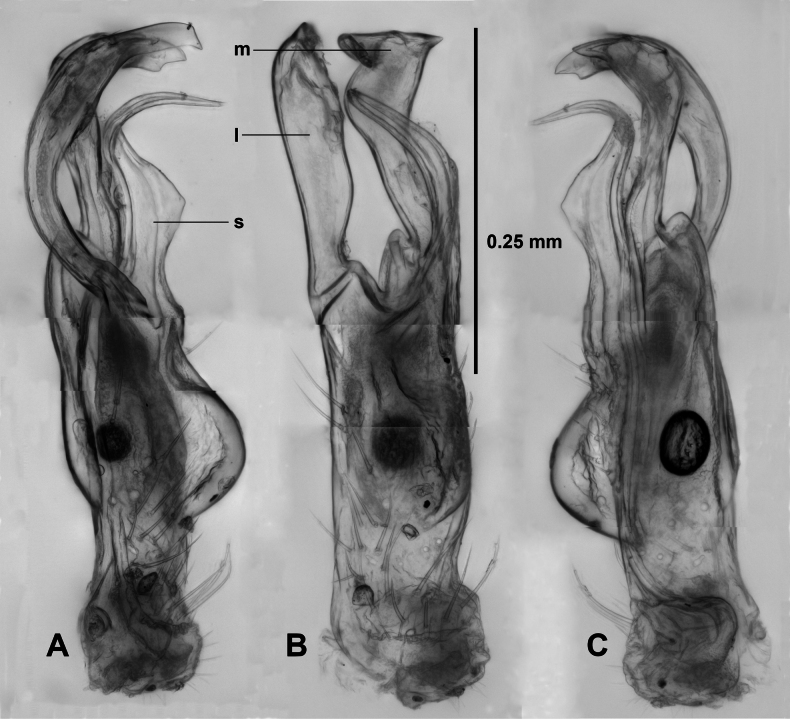
*Araneopedis porchi* sp. nov., paratype male ex site HS01-C-L2, NMV K16465. Lateral (**A**), posterior (**B**) and (**C**) medial views of cleared right gonopod telopodite; composite focus-stacked images. Abbreviations: l = lateral branch, m = medial branch, s = solenomere.

Telopodite (Fig. 1) with basal margin upturned. Telopodite erect, bulging posteriorly at ca 1/4 telopodite height, sparsely setose posteriorly at base and posterolaterally, divided at ca 1/2 telopodite height into lateral and medial branches of equal height. Lateral branch (Fig. 1) somewhat flattened anteroposteriorly, C-shaped with concavity on posterior side, tip directed posteriorly and bluntly pointed. Medial branch with rounded shoulder laterally at base, bent a little laterally before extending distally, widening slightly and bent posteriorly at apex, the tip with blunt tooth at lateral corner. Solenomere arising posterior to telopodite division, expanded posteriorly, curving laterally and flattening mediolaterally, the posterior edge forming a rounded triangle, then tapering strongly and bending posteriorly, the tip pointed posteriorly below lateral and medial branch tips. Prostatic groove running on medial surface of telopodite and along anterior edge of solenomere to open at solenomere tip.

##### Name.

Noun in the genitive case, in honour of Nicholas Porch (Deakin University), principal investigator for the Hermon Slade Foundation project in which this and other new millipede species were collected.

##### Distribution.

Collected from two locality clusters near Mt Hotham and Mt McKay in the Victorian mountains (Fig. 15A) with a linear range of ca 16 km, in alpine and subalpine grassland and woodland from 1725 to 1860 m. Possibly parapatric with *A. bogong* sp. nov.

##### Remarks.

**Figure 2.**
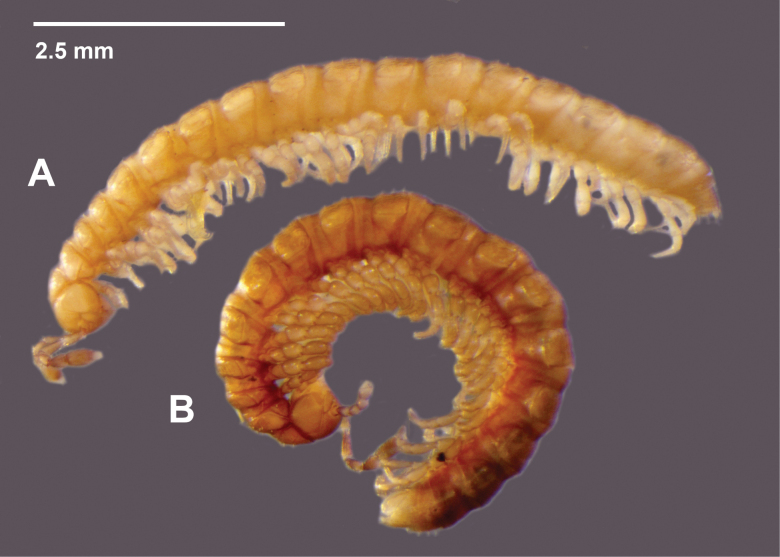
*Araneopedis porchi* sp. nov. males. **A.** Ex site HS02-O-L1, NMV K16467, litter extraction. **B.** Ex site HS02-O-P1, NMV K16478, pitfall trap.

Although the live colouring of *A. porchi* sp. nov. is currently unknown, specimens that were pitfall-trapped in propylene glycol are all almost uniformly darker brown (Fig. 2B). Propylene glycol may have stabilised colouring that was partially lost in the ethanol into which litter specimens were collected.

#### ﻿ Araneopedis bogong sp. nov.

Taxon classificationAnimaliaPolydesmidaDalodesmidae

CD79AFC2-667D-56B3-8C40-93F7D9FBD012

https://zoobank.org/E2EC364D-9014-4080-8F89-3931B501146F

Figs 3, 15A

##### Type material.

***Holotype*.** Male, Bogong High Plains (Vic), 0.37 km E by N of junction of Bogong High Plains Road and Big River Firetrail, site HS03-C-L2, -36.8725, 147.3218 ± 25 m, 1645–1665 m, coll. Nicholas Porch, 2024-04-22, 1 m^2^ litter sample from closed shrubby snow gum woodland, NMV K16482. ***Paratypes*.** 3F, details as for holotype, NMV K16481; 1M in 95% EtOH, same details but 0.2 km E by N of junction of Bogong High Plains Road and Big River Firetrail, site HS03-C-L1, -36.8730, 147.3199 ± 25 m, 1620–1640 m, NMV K16480; 1M, 8F, same details, NMV K16483; 1M, same details but Dinner Plain area, 0.51 km SSW of JB Plain carpark, site HS07-C-L3, -37.0264, 147.2187 ± 25 m, 1650 m, 2024-04-14, closed grassy/shrubby snow gum woodland, NMV K16484.

##### Additional material.

None.

##### Diagnosis.

Adult males distinguished from *Araneopedis buffalo* sp. nov. by the similar sizes of posterior rings vs enlarged rings; and from other species of *Araneopedis* gen. nov. by the solenomere flattened and with a wide distal-facing concavity.

##### Description.

As for the genus, with the following details. Male/female length ca 9.0/9.0 mm, maximum midbody width 0.8/0.8 mm, midbody metatergite width ca 1.4× prozonite width.

**Figure 3.**
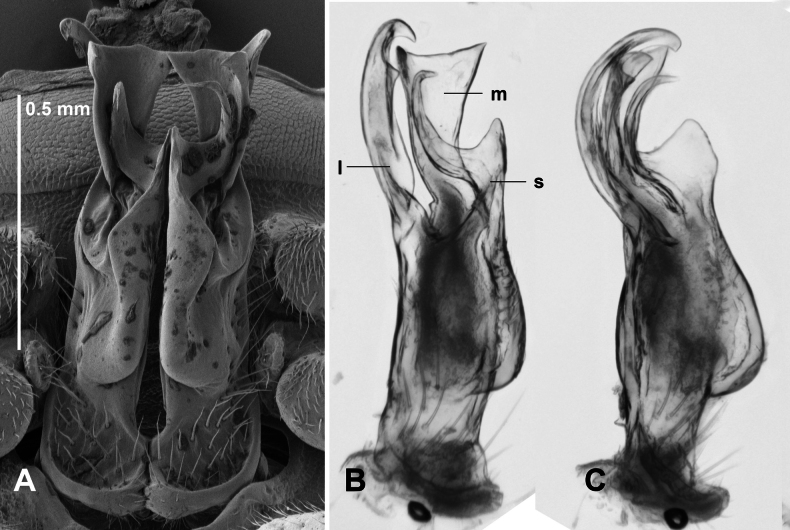
*Araneopedis bogong* sp. nov., paratype males ex site HS07-C-L3, NMV K16484 (**A**) and site HS03-C-L1, NMV K16483 (**B, C**). **A.** Posterior view of gonopods *in situ*; **B**, **C.** Medial and anteromedial views of cleared right gonopod telopodite; focus-stacked images. Abbreviations: l = lateral branch, m = medial branch, s = solenomere.

Telopodite base with upturned basal margin (Fig. 3A), sparsely setose. Division of telopodite into lateral and medial branches and solenomere at ca 1/2 telopodite height; telopodite bulging posteriorly below division. Lateral branch (Fig. 3B) a little taller than medial branch, narrow, flattened mediolaterally, bowed medially, tip curving posteriorly and pointed, lateral margin with triangular extension at ca 3/4 branch height. Medial branch widening from base and broadly truncate, concave posteriorly. Solenomere mediolaterally flattened, extending distally, then curving anterodistally, creating wide distal-facing concavity in profile, then tapering and curving posteriorly and terminating in posteriorly directed point with the opening of the prostatic groove.

##### Name.

Noun in apposition, for the Bogong High Plains, from which most of the known specimens were collected.

##### Distribution.

Collected from two locality clusters near Dinner Plain and east of Falls Creek in the Victorian mountains (Fig. 15A), a linear range of ca 19 km, in subalpine woodland from 1620 to 1665 m. Possibly parapatric with *A. porchi* sp. nov.

#### ﻿ Araneopedis buffalo sp. nov.

Taxon classificationAnimaliaPolydesmidaDalodesmidae

A2E037D6-3F2D-5B06-862D-DD30774DE1E2

https://zoobank.org/E086C259-3332-4ECA-8DC0-508CCD04C747

Figs 4, 5A, 15A

##### Type material.

***Holotype*.** Male, Mt Buffalo (Vic), 0.5 km SE by E of Mt Buffalo Park Office, site HS15-C-L1, -36.7281, 146.8079 ± 25 m, 1380–1400 m, coll. Nicholas Porch, 2024-04-13, 1 m^2^ litter sample from closed shrubby subalpine woodland, NMV K16486. ***Paratypes*.** 2M, 5F, details as for holotype but 0.3 km ESE of Cresta Valley carpark entrance, site HS13-O-L3, -36.7655, 146.7884 ± 25 m, 1465–1485 m, open herb-rich subalpine grassland, NMV K16488; 1M in 95% EtOH, same details, NMV K16485; 2M, 4F, same details but 0.29 km SE of Mt Buffalo Park Office, site HS15-C-L2, -36.7274, 146.8059 ± 25 m, 1370 m, closed shrubby subalpine woodland, NMV K16487.

##### Additional material.

2M from two other sites. See Suppl. material 1 for details.

##### Diagnosis.

Adult males distinguished from other species of *Araneopedis* gen. nov. by the posterior rings being enlarged vs of similar size to anterior rings (Fig. 5). Distinguished from *A. bogong* sp. nov. by the lack of a wide distal-facing concavity in the solenomere; from *A. porchi* sp. nov. and *A. dargo* sp. nov. by the terminal division of the lateral branch of the gonopod telopodite; and from *A. gibbae* sp. nov. by much larger size and presence of a terminal, posteriorly directed spine on the medial branch.

##### Description.

As for the genus, with the following details. Male/female length ca 9.0/8.5 mm, maximum midbody width 0.9/0.8, midbody metatergite width 1.3× prozonite width.

**Figure 4.**
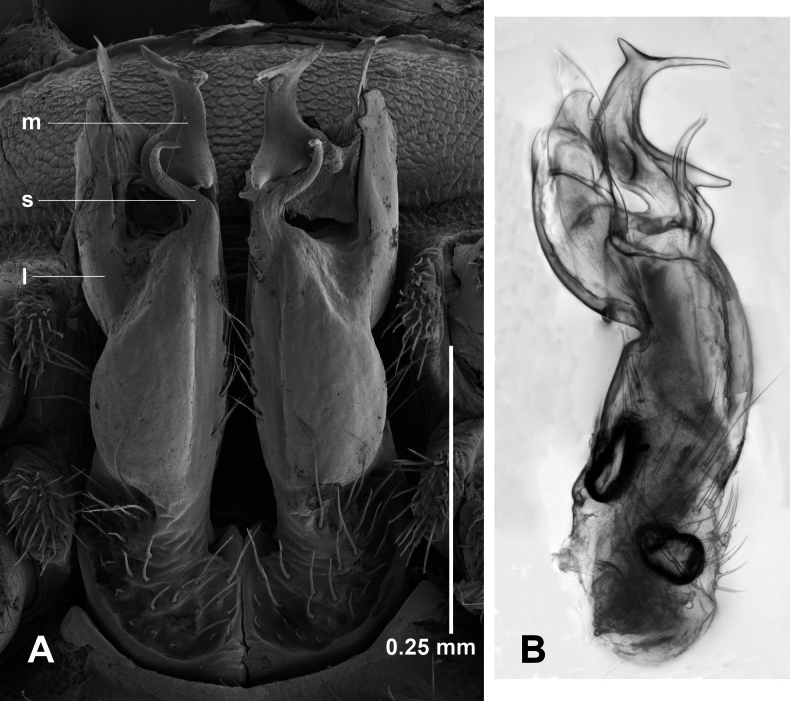
*Araneopedis buffalo* sp. nov., paratype males ex site HS15-C-L2, NMV K16487 (**A**), and site H13-O-L3, NMV K16488(**B**). **A.** Gonopods in situ; **B.** Lateral view of cleared right gonopod telopodite, composite focus-stacked image. Abbreviations: l = lateral branch, m = medial branch, s = solenomere.

Telopodite base with upturned basal margin (Fig. 4A), sparsely setose. Lateral branch arising lower on telopodite than medial branch + solenomere at ca 1/2 telopodite height, somewhat flattened mediolaterally, curving anterodistally, divided at ca 1/3 branch height into medial process and shorter lateral process with two posteriorly directed subapical teeth; medial process expanded medially as rounded tab anterior to medial branch tab, then extending distally with thin, mediolaterally flattened, pointed apex. Medial branch curving anterodistally and expanding medially as rounded tab with posteriorly directed spine arising medially on posterior surface; distal to expanded section curving posteriorly, apex truncate and extending anterolaterally as short blunt spine and posteromedially as longer, thinner spine. Lateral and medial branches reaching same height overall. Solenomere on posterior telopodite surface, thin and subcylindrical, curving first anteriorly, then distolaterally, the tip curving posterodistally; prostatic groove opening at end of tip. On anterior surface of telopodite, a mediolaterally flattened, subquadrate tab arising at level of medial branch origin, directed anteriorly.

**Figure 5.**
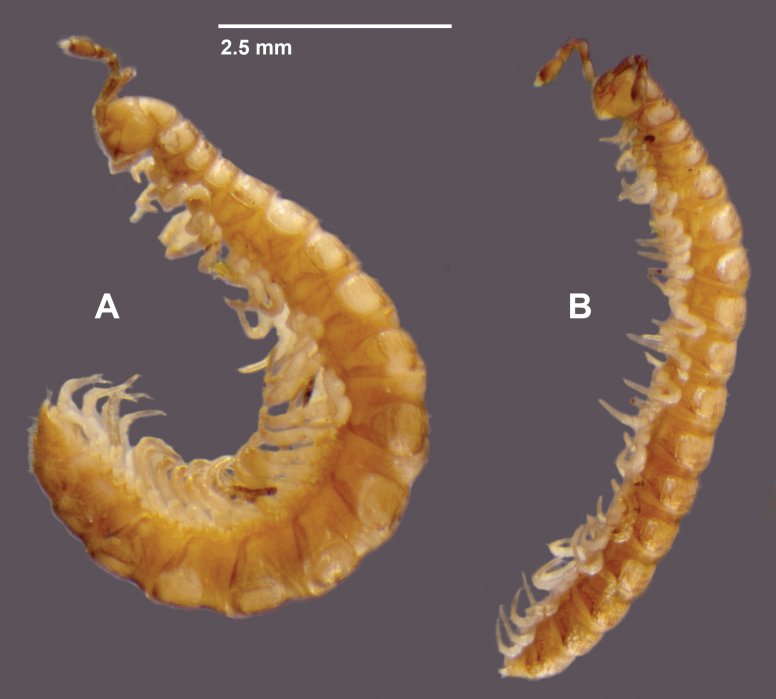
**A.***Araneopedis buffalo* sp. nov., holotype male ex site HS15-C-L1, NMV K16486; **B.***A. gibbae* sp. nov., holotype male ex site HS22-C-L3, NMV K16495.

##### Name.

Noun in apposition, for Mt Buffalo.

##### Distribution.

Collected near Mt Buffalo and Mt Buller in the Victorian mountains (Fig. 15A), in alpine and subalpine grassland and woodland from 1370 to 1740 m.

##### Remarks.

This species is very similar to *A. gibbae* sp. nov. in gonopod structure, but the male is substantially larger (Fig. 5A) and its posterior rings are proportionally larger than its anterior ones. The medial branch of the gonopod telopodite also differs from that of *A. gibbae* sp. nov. in having a terminal, posteriorly directed spine rather than a subterminal one. Females of this species are substantially smaller than males from the same site.

#### ﻿ Araneopedis dargo sp. nov.

Taxon classificationAnimaliaPolydesmidaDalodesmidae

E5F9FEC0-8C04-5290-B5F4-4C8C0F3173B3

https://zoobank.org/B3CADD8B-BFC5-4429-9F49-C823BCBD8A69

Figs 6, 15A

##### Type material.

***Holotype*.** Male, Dargo High Plains (Vic), King Spur Track, E of Dargo High Plains Road, site HS11-O-L2, -37.1151, 147.1782 ± 25 m, 1525–1545 m, coll. Nicholas Porch, 2024-04-14, 1 m^2^ litter sample from open shrubby subalpine grassland, NMV K16492. ***Paratypes*.** 3M, 7F, details as for holotype, NMV K16494; 1M in 95% EtOH, same details, NMV K16493; 2M, 16F, same details but site HS11-O-L3, -37.1162, 147.1788 ± 25 m, open grassy subalpine shrubland NMV K16491.

##### Additional material.

None.

##### Diagnosis.

Adult males distinguished from *A. buffalo* sp. nov. by the posterior rings being of similar size to anterior rings vs enlarged; and from *A. buffalo* sp. nov. and other species of *Araneopedis* gen. nov. by the medial branch of the gonopod telopodite being much taller than the lateral branch.

##### Description.

As for the genus, with the following details. Male/female length ca 8.5/8.0 mm, maximum midbody width 0.75/0.7.5 mm, midbody metatergite width ca 1.4× prozonite width.

**Figure 6.**
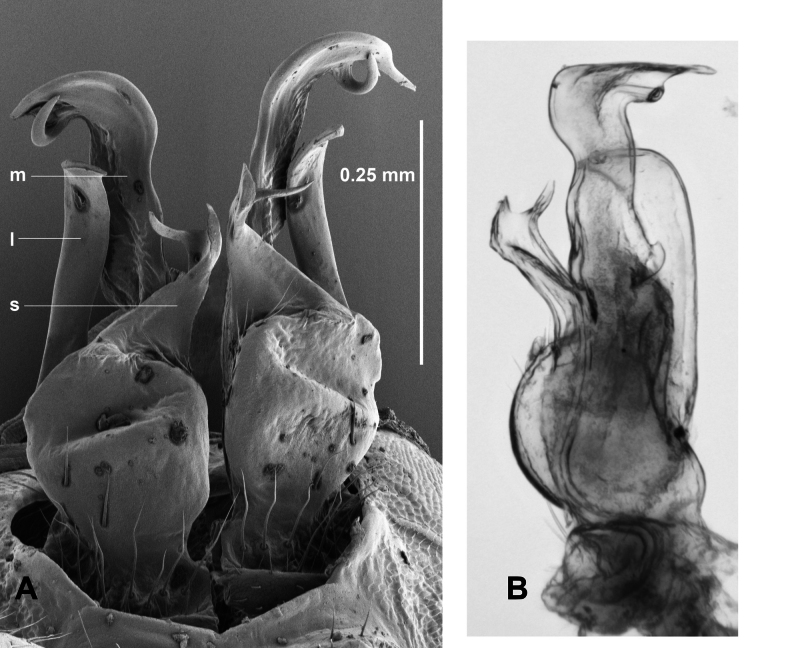
*Araneopedis dargo* sp. nov., paratype males ex site HS11-O-L2, NMV K16494. **A.** Gonopods *in situ* (bulbous base of telopodite has collapsed due to drying); **B.** Medial view of cleared right gonopod telopodite; focus-stacked image. Abbreviations: l = lateral branch, m = medial branch, s = solenomere.

Telopodite base with upturned basal margin (Fig. 6A, B), sparsely setose with long setae. Proximal portion of telopodite bulbous, expanded posteriorly. Lateral branch arising near telopodite base, extending distally as strap-like structure with near-constant width, tilted medially to stand just posterior to medial branch, the tip curving posteriorly and bluntly pointed. Medial branch similarly strap-like but thicker and longer than lateral branch, somewhat sinuous in outline, the apex bent laterally and tapering to blunt point distal to tip of lateral branch; subapically on anterolateral edge with blunt, spine-projection tightly curving posterodistally. Solenomere arising from top of bulbous telopodite section at ca 1/2 telopodite height, directed anteromedially, flattened, the truncate distal edge with short pointed tooth posteriorly and longer, spine-like extension curving distolaterally and terminating with opening of prostatic groove. Prostatic groove running along anterior edge of solenomere.

##### Name.

Noun in apposition, for the Dargo High Plains.

##### Distribution.

Collected on the Dargo High Plains in the Victorian mountains (Fig. 15A), in subalpine grassland and shrubland from 1525 to 1545 m.

#### ﻿ Araneopedis gibbae sp. nov.

Taxon classificationAnimaliaPolydesmidaDalodesmidae

91551C21-BD88-5E86-8418-7B8849829B65

https://zoobank.org/8CDBFE81-1A6D-4C96-89D6-B8AB1979A673

Figs 5B, 7, 15A

##### Type material.

***Holotype*.** Male, Howitt Plains (Vic), 0.19 km W by N of Mount Howitt carpark, site HS22-C-L3, -37.2045, 146.6779 ± 25 m, 1610–1630 m, coll. Nicholas Porch and Heloise Gibb, 2024-03-01, 1 m^2^ litter sample from closed shrubby subalpine woodland, NMV K16495. ***Paratypes*.** 3M, 3F, details as for holotype, NMV K16497; 1M in 95% EtOH, same details, NMV K16496.

##### Additional material.

None.

##### Diagnosis.

Adult males distinguished from *A. buffalo* sp. nov. by the posterior rings being of similar size to anterior rings vs enlarged, and by the much smaller body size; from *A. dargo* sp. nov. by the lateral and medial branches of the gonopod telopodite being equally tall vs lateral branch much shorter than medial branch; from *A. porchi* sp. nov. by the divided vs undivided lateral branch; and from *A. bogong* sp. nov. by the lack of a wide distal-facing concavity in the solenomere.

##### Description.

As for the genus, with the following details. Male/female length ca 7/7 mm, maximum midbody width 0.6/0.7 mm, midbody metatergite width ca 1.3× prozonite width.

**Figure 7.**
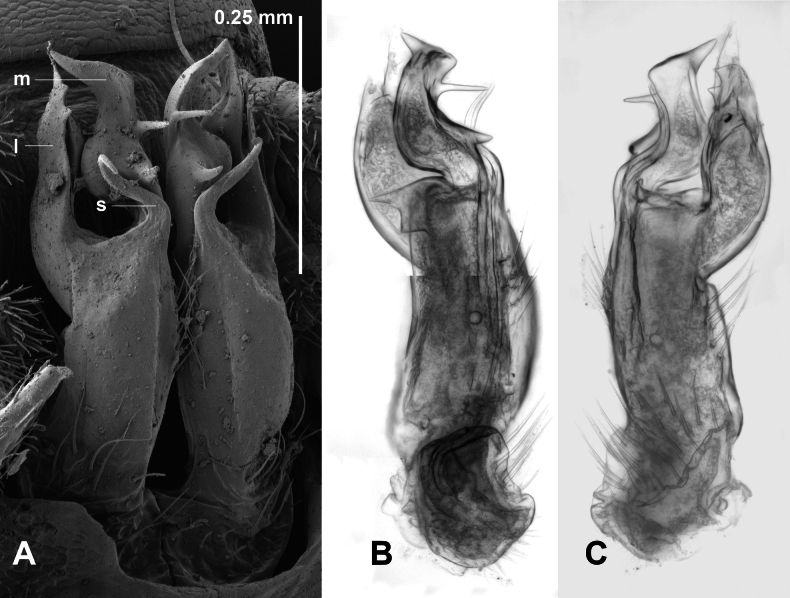
*Araneopedis gibbae* sp. nov., paratype males ex site HS22-C-L3, NMV K16497. **A.** Gonopods in situ; **B**, **C.** Cleared left gonopod telopodite in medial (**B**) and lateral (**C**) views; composite focus-stacked images. Abbreviations: l = lateral branch, m = medial branch, s = solenomere.

Telopodite base with upturned basal margin (Fig. 7A), sparsely setose. Lateral branch arising lower on telopodite than medial branch + solenomere at ca 1/2 telopodite height, somewhat flattened mediolaterally, curving anterodistally, divided at ca 1/2 branch height into medial process and shorter lateral process with two posteriorly directed subapical teeth; medial process expanded medially as rounded tab anterior to medial branch tab, then extending distally with thin, mediolaterally flattened, pointed apex. Medial branch extending distally, then curving anterodistally and expanding into bulbous section with posteriorly directed spine on posterior surface; distal to bulbous section tapering and curving laterally, with a second posteriorly directed spine on posterior surface. Lateral and medial branches reaching same height overall. Solenomere on posterior telopodite surface, thin and subcylindrical, curving first anteriorly, then distolaterally, the tip curving posterodistally; prostatic groove opening at end of tip. On anterior surface of telopodite, a mediolaterally flattened, subquadrate tab arising at level of medial branch origin, directed anteriorly with distal corner projecting.

##### Name.

Noun in the genitive case, in honour of Heloise Gibb (Deakin University), co-collector of this species. Gibb is a community ecologist with a strong interest in invertebrate conservation.

##### Distribution.

Collected near Mt Howitt in the Victorian mountains (Fig. 15A), in subalpine woodland from 1610 to 1630 m.

##### Remarks.

This species is very similar to *A. buffalo* sp. nov. in gonopod structure, but the male is substantially smaller (Fig. 5A, B) and the posterior rings are not proportionally larger than the anterior rings. The medial branch of the gonopod telopodite also differs from that of *A. buffalo* sp. nov. in having a subterminal, posteriorly directed spine rather than a terminal one.

#### ﻿Genus Cernethia

Taxon classificationAnimaliaPolydesmidaDalodesmidae

Mesibov, 2015

83AD387D-8F85-5DCF-849B-FA530EE42843

Cernethia Mesibov 2015: 142.

##### Type species.

*Cernethia inopinata* Mesibov, 2015 by original designation.

##### Other included species.

*C. dysmica* sp. nov.

#### ﻿ Cernethia dysmica sp. nov.

Taxon classificationAnimaliaPolydesmidaDalodesmidae

DB2244F3-3214-5C26-B739-3AA3459AAE97

https://zoobank.org/D57625E4-5770-4BAF-BF47-B3564A6DDB63

Figs 8, 15B

##### Type material.

***Holotype*.** Male, Kosciuszko National Park (NSW), 0.34 km W of summit of Mt Guthrie, site HS39-C-L2, -36.4280, 148.3334 ± 25 m, 1880 m, 2025-02-09, coll. Nicholas Porch and Aidan Fitt, 1 m^2^ litter sample from shrubby subalpine snow gum woodland, NMV K16501. ***Paratypes*.** 1M, 5F, 11J, same details, NMV K16502; 2M, 1F, same details but 0.31 km W by N of summit of Mt Guthrie, site HS39-C-L1, -36.4275, 148.3338 ± 25 m, 1875 m, NMV K15990.

##### Additional material.

2M, 6F, 10J from four other sites. See Suppl. material 1 for details.

##### Diagnosis.

Solenomere with medial portion directed anteromedially in *Cernethia dysmica* sp. nov., directed distally in *C. inopinata*; small, finger-like, posterobasally directed process near telopodite division in *C. inopinata* lacking in *C. dysmica* sp. nov. Medial process of telopodite more or less straight in *C. inopinata*, sinuous in *C. dysmica* sp. nov.

##### Description.

**Figure 8.**
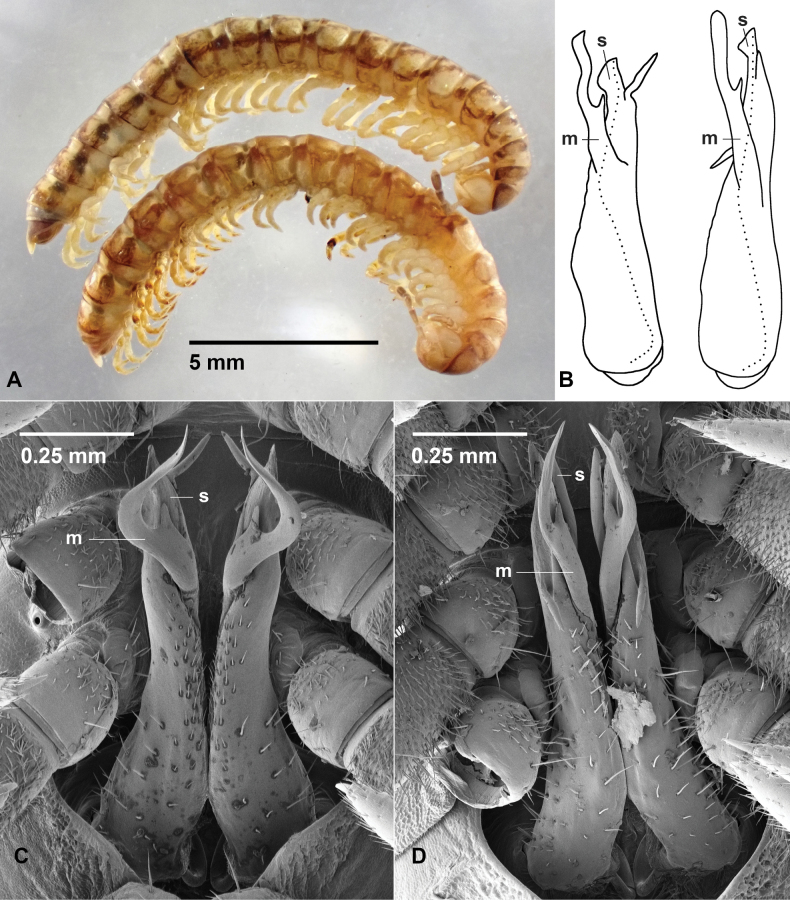
*Cernethia dysmica* sp. nov. **A.** Lower, paratype male ex site HS39-C-L2, NMV K16502; **B.** Left; **C.** Paratype male ex site HS39-C-L1, NMV K15990) and *C. inopinata* (**A.** Top; **B.** Right; **D.** Ex site HS 53-C-L3, NMV K15993). **A.** Males preserved in alcohol; **B.** Outline sketches of right gonopod telopodite in medial view, with dotted line marking path of prostatic groove; **C**, **D.** Ventral views of gonopods in situ. Abbreviations: m = medial branch, s = solenomere.

Males and females as for *C. inopinata*, including colour pattern in alcohol (Fig. 8A); length ca 14–15 mm. Gonopod telopodite (Fig. 8B left, C) erect, subcylindrical, tapering from base, divided at ca 1/2 telopodite height into mediolaterally flattened, lateral solenomere and medial branch. Solenomere divided near apex; lateral portion carrying prostatic groove, medial portion short, finger-like, directed anteromedially; lateral portion with very small spine-like process directed posterolaterally, sometimes broken off; solenomere tip rounded and slightly expanded posteriorly. Medial branch of telopodite flattened mediolaterally, directed distolaterally, then sinuously curving distomedially, with small tooth on anterior edge at ca 1/3 branch height; medial branch terminating just distal to solenomere tip. Prostatic groove on medial surface of telopodite, running between telopodite branches and following solenomere to tip.

Female a little shorter than male, stouter and with thinner legs; epigynum elevated medially; cyphopods not examined.

##### Distribution.

Collected in subalpine woodland at 1590 to 1880 m, at several localities from Dead Horse Gap to Rennix Gap in Kosciuszko National Park, NSW, a linear range of ca 20 km (Fig. 15B).

##### Name.

Adjective from Greek *dysmikos*, “western”. This species was found west of the type locality for *C. inopinata*.

##### Remarks.

The two *Cernethia* species are very similar and can only be distinguished by inspection of the gonopod telopodite of a mature male. Like *C. inopinata*, *C. dysmica* sp. nov. has a powerful defensive secretion whose smell persists in alcohol-preserved material.

#### ﻿ Dibologonus gen. nov.

Taxon classificationAnimaliaPolydesmidaDalodesmidae

388F578E-6AD9-51F3-A3F6-A86D716C88B7

https://zoobank.org/369D7AEA-A5AF-4FBA-886B-CB3AB1DBAB99

##### Type species.

*Dibologonus sladei* sp. nov., by present designation.

##### Other assigned species.

*Dibologonus major* sp. nov., *D. minor* sp. nov., *D. oedipus* sp. nov.

##### Diagnosis.

Distinguished from other described Australian dalodesmids by the very slender gonopod telopodite split into a medial branch and a lateral solenomere with a helical prostatic groove; the two branches straight, extending distally in parallel and close together.

##### Description.

Males and females with body plan head+19 rings (including telson).

Male with vertex sparsely setose, frons and clypeus setose; vertigial sulcus reaching 1/2 way to top of antennal sockets; sockets separated by ca 2× socket diameter. Relative ring widths: 6 > 5 > 4 > 3 > 2 > collum; ring widths 6–13 subequal, diminishing posteriorly; head approximately as wide as ring 4 or 5. Collum half-moon-shaped in dorsal view, posteriorly slightly emarginate, corner bluntly rounded. Tergite 2 without ventral pit; paranotal margin a little lower than tergite 3 margin. Midbody paranotal margin at ca 1/2 ring height, level. Paranotum with anterior corner rounded, laterally gently convex, posterior corner not or only slightly projecting. Midbody ring with metatergite width ca 1.3–1.4× prozonite width; waist well-defined. Metatergite nearly smooth, with 2 transverse rows and a posterior marginal row of very small setae. Pore formula normal, ozopore near posterior corner of paranotum. Limbus a simple row of spine-like elements. Spiracles small, round, barely raised above pleurite surface, on diplosegments with posterior spiracle between leg bases. Sternites a little longer than wide, transverse impression deeper and wider than longitudinal impression, variably setose. Relative podomere lengths of midbody leg: tarsus > femur > prefemur > (postfemur, tibia); tarsus straight. Epiproct short, conical; hypoproct subtrapezoidal or broadly paraboloid; spinnerets in square array.

Gonopore small, opening distomedially on leg 2 coxa. Gonocoxae small, subconical, lightly joined distomedially. Aperture a little longer than wide, ca 1/2 ring 7 prozonite width, margin produced posterolaterally. Gonopod telopodites slender, straight, split into medial branch and lateral solenomere; ring 6 sternite excavate to accommodate retracted telopodites, with small tufts of setae above leg bases. Prostatic groove running from medial surface at telopodite base across posterior surface, then between medial branch and solenomere and curving in spiral around solenomere (left-handed spiral on left telopodite, right-handed spiral on right telopodite).

##### Name.

Greek *dibolos* (“two-pointed”) plus “-gonus”, an ending for genus names in Polydesmida that refers to gonopod structure. The gonopod telopodite is split into two parallel processes. Gender masculine.

##### Remarks.

**Figure 9.**
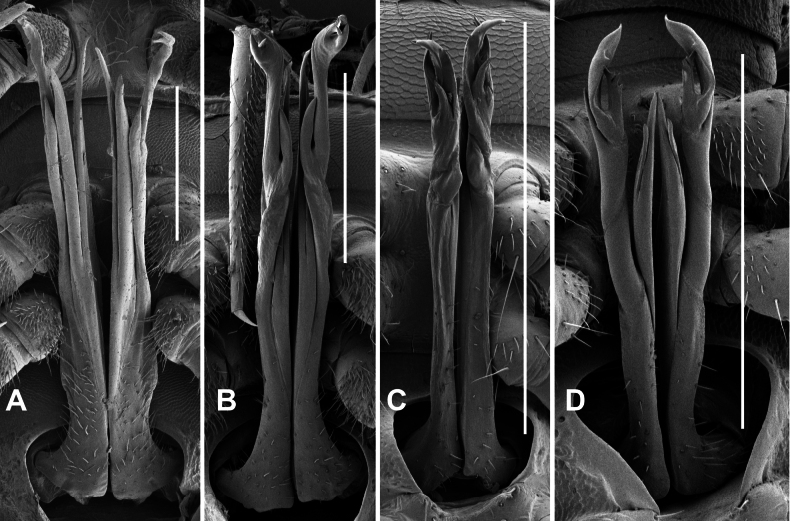
*Dibologonus* gen. nov., gonopods *in situ*. **A.***D. sladei* sp. nov., paratype ex site HS10-C-L3, NMV K16005; **B.***D. major* sp. nov., paratype ex site NP23-18, NMV K16010; **C.***D. minor* sp. nov., paratype ex site NP23-11, NMV K16505; **D.***D. oedipus* sp. nov., paratype ex site HS40-C-L2, NMV K16526. Scale bars: 0.5 mm.

The four species of *Dibologonus* gen. nov. are partly distinguishable by adult size, but more reliably by differences in the structure of the gonopod telopodite. As the telopodite processes are tightly packed together, these differences are best seen under high magnification (Fig. 9).

Species of *Dibologonus* gen. nov. occur widely in eastern Victoria and southeastern New South Wales (Fig. 16A), and more species (or significant range extensions) are likely to be found in future.

#### ﻿ Dibologonus sladei sp. nov.

Taxon classificationAnimaliaPolydesmidaDalodesmidae

2D992188-62B7-5E58-BF3E-964194519223

https://zoobank.org/ED34DE1A-DB54-4AB4-97B5-02C79F503081

Figs 9A, 10, 16A

##### Type material.

***Holotype*.** Male, Dargo High Plains Road (Vic), 90 m S by W of King Spur Track junction, site HS10-C-L3, -37.1134, 147.1662 ± 25 m, 1575–1595 m, coll. Nicholas Porch, 2024-04-14, 1 m^2^ litter sample from closed snow gum woodland, NMV K15999. ***Paratypes*.** 13M, 24F, details as for holotype, NMV K16005; 6M, 14F, same details but 0.14 km SE by E of King Spur Track junction, site HS10-C-L1, -37.1134, 147.1677 ± 25 m, 1565–1585 m, NMV K16002; 1M, same details but in 95% EtOH, NMV K15998; 12M, 13F, same details but 0.13 km S by E of King Spur Track junction, site HS10-C-L2, -37.1137, 147.1669 ± 25 m, 1570–1590 m, NMV K16004.

##### Additional material.

11M, 15F from five other sites. See Suppl. material 1 for details.

##### Diagnosis.

Much larger than *D. minor* sp. nov. and *D. oedipus* sp. nov. Solenomere with apex not expanded as in *D. major* sp. nov. and *D. oedipus* sp. nov.; major telopodite division into medial branch and solenomere at ca 1/3 telopodite height vs 2/3 height as in *D. minor* sp. nov.

##### Description.

**Figure 10.**
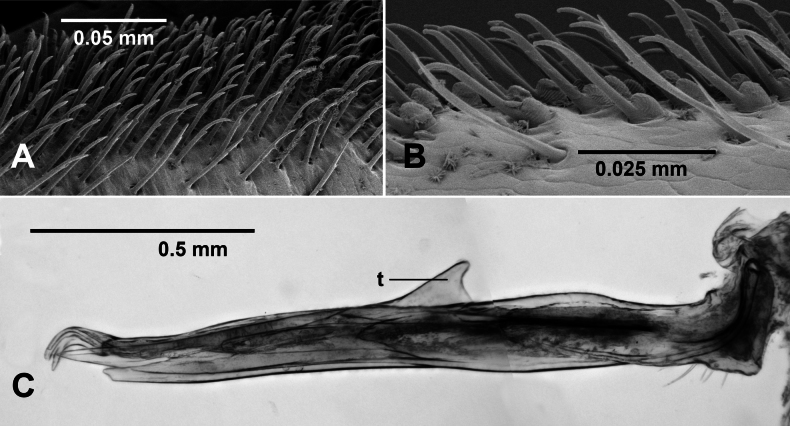
*Dibologonus sladei* sp. nov. **A**, **B.** Paratype male ex site HS10-C-L3, NMV K16005; **C.** paratype male ex site HS10-C-L1, NMV K16002; **A.** Leg 4 brush setae on prefemur (distal to right); **B.** Leg 4 sphaerotrichomes on tarsus (distal to left); **C.** Medial view of cleared right gonopod telopodite; focus-stacked image. Abbreviation: t = anterior tab.

As for the genus, with the following details. Colour in alcohol brownish-yellow with pale brown at collum and paranotal margins. Adult male/female approximate measurements: length 14/14 mm, maximum midbody width 1.5/1.5 mm. Brush setae dense, tips curving distally and with mid-height notch on distal surface (Fig. 10A); sphaerotrichomes with slightly flattened globular base, shafts with rounded tips curving distally (Fig. 10B).

Gonopod telopodites (Figs 9A, 10C) almost reaching legpair 4 when retracted, setose posterolaterally to ca 1/3 telopodite height. Telopodite divided into medial branch and solenomere at ca 1/3 telopodite height. Medial branch divided at ca 1/2 telopodite height into parallel anteromedial and posterolateral processes, both processes somewhat flattened; anteromedial process with blunt, rounded tip below level of solenomere; posteromedial process shorter than anteromedial, with truncate tip. Solenomere divided at ca 3/4 telopodite height into anterolateral process and much shorter, parallel, posteromedial process, the latter somewhat flattened and tapering to point. Anterolateral process somewhat flattened, curving a little laterally, bending medially at apex and dividing into two short, tooth-like processes, the prostatic groove opening on the proximal tooth. On the anterior surface of the telopodite at ca 1/2 telopodite height, a prominent triangular tab (Fig. 10C) with apex directed anterobasally.

Female approximately the same length as male but a little stouter and with thinner legs. Epigynum raised medially in low, rounded triangle; cyphopods not examined.

##### Name.

Noun in the genitive case, in honour of George Hermon Slade (1910–2002), Australian chemist, orchidologist and founder of the Hermon Slade Foundation for support of research in the natural sciences.

##### Distribution.

Collected over a ca 55 km linear range from Mt Buffalo National Park to the Dargo High Plains (Fig. 16A), in subalpine woodland from 1500 to 1640 m.

##### Remarks.

None of the males examined have modified legs as seen in *D. oedipus* sp. nov.

#### ﻿ Dibologonus major sp. nov.

Taxon classificationAnimaliaPolydesmidaDalodesmidae

A5EFEFE2-4170-5912-AB4E-83AA367B3FAB

https://zoobank.org/E4E92624-6A4A-492E-8486-3BB3FE9EEB08

Figs 9B, 11, 16A

##### Type material.

***Holotype*.** Male, Baw Baw National Park (Vic), 0.5 km NNE of Mt St Gwinear carpark, site NP23-18, -37.8368, 146.3346 ± 25 m, 1270 m, coll. Nicholas Porch, 2023-03-15, 2 m^2^ litter sample from riparian cool temperate rainforest, NMV K16008. ***Paratypes*.** 2M, details as for holotype, NMV K16010; 1M in 95% EtOH, same details but Mt St Gwinear Road, 1.1 km WNW of junction with Thomson Valley Road, site NP23-20, -37.8163, 146.3456 ± 25 m, 1035 m, wet sclerophyll forest, NMV K16009.

##### Additional material.

None.

##### Diagnosis.

Much larger than *D. minor* sp. nov. and *D. oedipus* sp. nov. Solenomere with apex expanded vs not expanded in *D. sladei* sp. nov.; the major telopodite division into medial branch and solenomere at ca 1/3 telopodite height vs 2/3 height in *D. minor* sp. nov.; solenomere obscuring medial branch vs well-separated from medial branch in *D. oedipus* sp. nov.

##### Description.

**Figure 11.**
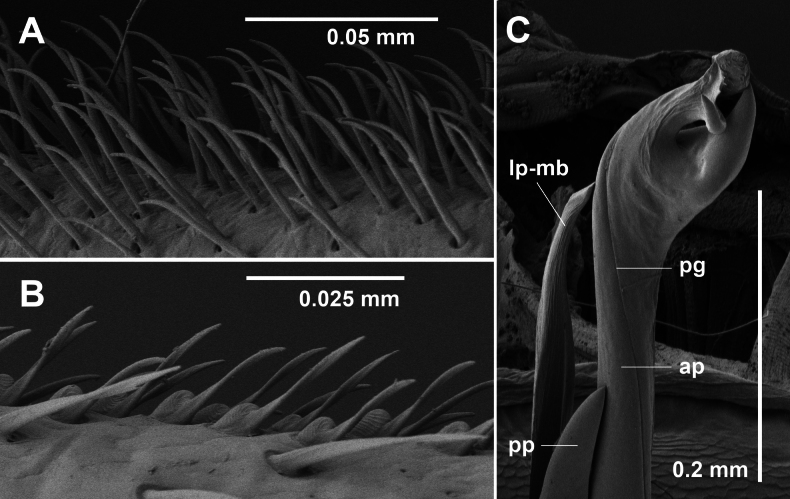
*Dibologonus major* sp. nov., paratype male ex site NP23-18, NMV K16010. **A.** Leg 6 brush setae on prefemur (distal to left); **B.** Leg 6 sphaerotrichomes on tarsus (distal to right); **C.** Distal portion of left gonopod telopodite. Abbreviations: ap = anterolateral process of solenomere, lp-mb = lateral process of medial branch, pg = prostatic groove, pp = posteromedial process of solenomere, t = tooth with opening of prostatic groove.

As for the genus, with the following details. Male colour in alcohol pale yellow, sometimes with fine reddish-brown speckling at collum and paranotal margins. Length ca 13 mm, maximum midbody diameter 1.6 mm. Antenna reaching tergite 3 when manipulated dorsally; relative antennomere lengths 3 > (2,6) > (4,5); 6 widest. Metatergites with posterior corners projecting a little, more on rings 14–17. Midbody leg ca 1.4× ring diameter. Brush setae (Fig. 11A) dense, tapering to point curving distally on coxa/trochanter, prefemur, femur, with a few on postfemur, without mid-height notch; sphaerotrichomes (Fig. 11B) on postfemur, tibia and tarsus with flattened globular base and tapering shaft, the shaft declined distally and flattened.

Gonopod telopodites (Figs 9B, 11C) straight, almost reaching legpair 4 when retracted, setose posterolaterally to ca 1/3 telopodite height. Telopodite divided at ca 1/2 height into medial branch and solenomere. Medial branch gradually tapering, divided near base into medial and lateral processes tapering to points, the lateral process a little longer. Solenomere gently curving medially near base, divided at ca 3/4 telopodite height into posteromedial and anterolateral processes. Posteromedial process stout, pointed, terminating below other telopodite processes. Anterolateral process subcylindrical, expanding near tip and divided into a stout, pointed, lateral subprocess and a medial subprocess curving medially towards the lateral subprocess tip, and with small subapical tooth curving posterodistally and carrying the termination of the prostatic groove.

##### Name.

Adjective; Latin *major*, “greater”. This is the larger of the two species of *Dibologonus* gen. nov. co-occurring near Mt Baw Baw.

##### Distribution.

Collected at two sites ca 2.5 km apart near Mt St Gwinear (Fig. 16A), in wet sclerophyll forest at 1035 m and cool temperate rainforest at 1270 m. Co-occurs with *D. minor* sp. nov.

##### Remarks.

No females of this species have yet been recognised, and none of the four males examined have modified legs as seen in *D. oedipus* sp. nov.

#### ﻿ Dibologonus minor sp. nov.

Taxon classificationAnimaliaPolydesmidaDalodesmidae

649CFDA3-0B94-5C30-9ABC-BE4BC1192382

https://zoobank.org/189FABEC-250B-44D3-9A84-E83FFF10A8F7

Figs 9C, 12, 16A

##### Type material.

***Holotype*.** Male in 95% EtOH, Mt Baw Baw, 0.43 km S by W of Mt Baw Baw summit, site HS20-C-L3, -37.8433, 146.2748 ± 25 m, 1410–1430 m, 2024-02-13, coll. Nicholas Porch and Daniel Kurek, 1 m^2^ litter sample from closed shrubby subalpine woodland, NMV K16012. ***Paratypes*.** 1M, 2F, Baw Baw Tourist Road, 0.79 km WNW of Baw Baw Alpine Resort entrance, site NP23-11, -37.8475, 146.2324 ± 25 m, 760 m, 2023-03-05, coll. Nicholas Porch, 2 m^2^ litter sample from snow gum woodland, NMV K16505; 2M, same details but Baw Baw Alpine Reserve, near NE middle of carpark 3, site NP23-05, -37.8382, 146.2611 ± 25 m, 1460 m, NMV K16503; 1F, same details but Baw Baw Alpine Reserve, 40 m N of Saxons Picnic Area, site NP23-08, -37.8354, 146.2589 ± 25 m, 1435 m, NMV K16011; 1M, 2F, same details but Baw Baw Alpine Reserve, 0.1 km NE by E of Saxons Picnic Area, site NP23-10, -37.8352, 146.2599 ± 25 m, 1430 m, NMV K16504; 1M, same details but Baw Baw National Park, 0.5 km NNE of Mt St Gwinear carpark, site NP23-18, -37.8368, 146.3346 ± 25 m, 1270 m, 2023-03-15, NMV K16013; 2M, same details but Mt Baw Baw Alpine Reserve, 0.35 km W of Mt Baw Baw summit, site HS27-O-L3, -37.8371, 146.2734 ± 25 m, 1560 m, coll. Nicholas Porch and Aidan Fitt, grassy/shrubby snow gum woodland, NMV K16014.

##### Additional material.

None.

##### Diagnosis.

Much smaller than *D. sladei* sp. nov. and *D. major* sp. nov. Distinguished from the other three species of *Dibologonus* gen. nov. in having the major division into medial branch and solenomere at ca 2/3 telopodite height vs 1/3 height.

##### Description.

As for the genus, with the following details. Colour in alcohol pale yellow, sometimes with fine reddish-brown speckling at collum and paranotal margins. Adult male/female approximate measurements: length 8/9 mm, maximum midbody diameter 0.8/0.9.

**Figure 12.**
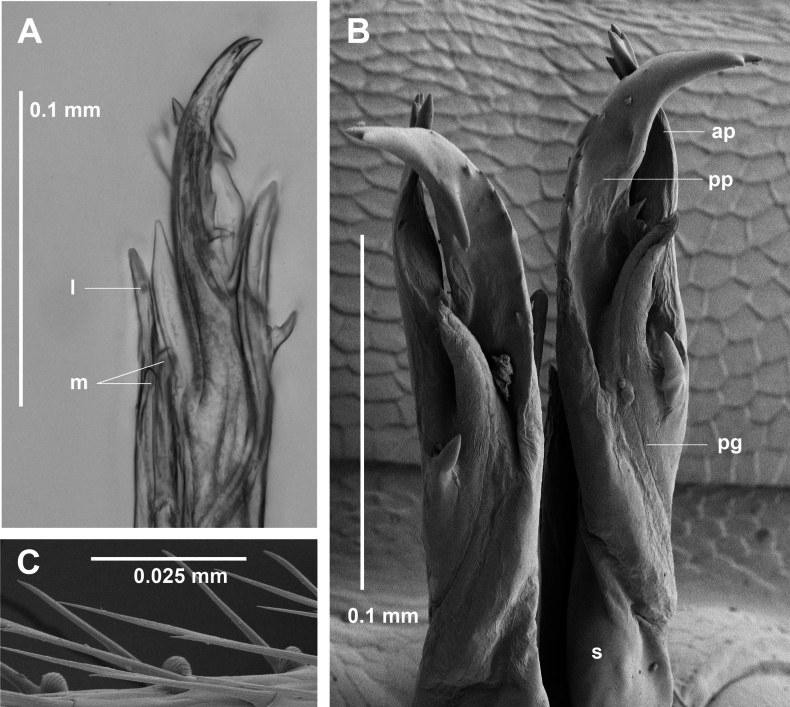
*Dibologonus minor* sp. nov., paratype males. **A.** Ex site HS27-O-L3, NMV K16014; **B.** Ex site NP23-11, NMV K16505. **A.** Focus-stacked medial view of distal end of cleared left gonopod telopodite; **B.** Posterior view of distal ends of gonopod telopodites; **C.** Sphaerotrichomes on leg 4 tarsus. Abbreviations: ap = anterolateral process, l = lateral process, m = the two medial process divisions of medial branch, pg = prostatic groove, pp = posteromedial process, s = solenomere.

Male with antenna almost reaching tergite 3 when manipulated dorsally; relative antennomere lengths 6 > 3 > 2 > (4,5); 6 widest. Metatergites slightly wider posteriorly, midbody metatergite width ca 1.2× prozonite width. Midbody leg ca 1.4× ring diameter. Brush setae tapering to point curving a little proximally, with mid-height notch as in *D. sladei* sp. nov. on coxa/trochanter, prefemur, femur; sphaerotrichomes (Fig. 12C) on postfemur, tibia and tarsus with very small globular base and pointed shaft, the tip not curving.

Gonopod telopodites (Figs 9C, 12A, B) straight, almost reaching legpair 5 when retracted, sparsely setose posterolaterally to ca 1/2 telopodite height. Telopodite divided at ca 2/3 telopodite height into medial branch and solenomere. Medial branch divided near base into medial and lateral processes, the medial process shorter and further divided into parallel, bluntly pointed subprocesses; medial branch hidden in posterior view by solenomere. Solenomere divided at ca 3/4 telopodite height into anterolateral process and posteromedial process, with small rounded tooth on posterior telopodite surface near division. Anterolateral process flattened, curving slightly medially near tip, the apex with a cluster of four or five small teeth. Posteromedial process with small, horn-like, posterior subprocess curving laterally, the prostatic groove terminating at tip; posteromedial process then extending distally as flattened subprocess, becoming cylindrical and curving posterolaterally, with minute teeth on margins and at apex, and with small, basally directed tooth above horn-like subprocess.

Female a little larger than male, epigynum subrectangular; cyphopods not examined.

##### Name.

Adjective; Latin *minor*, “less”. This is the smaller of the two species of *Dibologonus* gen. nov. co-occurring near Mt Baw Baw.

##### Distribution.

Collected over a ca 9 km linear range near Mt Baw Baw (Fig. 16A), in subalpine woodland and riparian rainforest from 760 to 1560 m. Co-occurs with *D. major* sp. nov.

##### Remarks.

None of the males examined have modified legs as seen in *D. oedipus* sp. nov.

#### ﻿ Dibologonus oedipus sp. nov.

Taxon classificationAnimaliaPolydesmidaDalodesmidae

1BB54D62-97A4-5117-9E4A-249587F21CB7

https://zoobank.org/853C5D0A-A66E-40B8-9F8E-835F8486CAB0

Figs 9D, 13, 16A

##### Type material.

***Holotype*.** Male, Kosciuszko National Park (NSW), 0.49 km NE of Rennix Gap, site HS41-O-L3, -36.3579, 148.5060 ± 25 m, 1580 m, coll. Nicholas Porch, 2025-02-22, 1 m^2^ litter sample from open shrubby subalpine grassland, NMV K16511. ***Paratypes*.** 8M, 17F, details as for holotype, NMV K16516; 7M, 2F, same details but 0.58 km NNE of Rennix Gap, site HS40-C-L2, -36.3561, 148.5046 ± 25 m, 1600 m, coll. Nicholas Porch and Aidan Fitt, 2025-02-09, closed shrubby subalpine woodland, NMV K16526; 1M, same details but in 95% EtOH, NMV K16510; 9M, 1F, same details but 0.52 km NNE of Rennix Gap, site HS40-C-L3, -36.3579, 148.5060 ± 25 m, 1595 m, NMV K16506.

##### Additional material.

16M, 33F, 3J from 16 other sites. See Suppl. material 1 for details.

##### Diagnosis.

Smaller than *D. sladei* sp. nov. and *D. major* sp. nov. Distinguished from *D. minor* sp. nov. in having the major telopodite division at 1/3 telopodite height vs 2/3 height; distinguished from *D. sladei* sp. nov. by the expanded vs non-expanded apex of the solenomere; distinguished from *D. major* sp. nov. by the solenomere being well-separated from the medial branch, not obscuring it. In most male specimens, distinguished from *D. sladei* sp. nov., *D. major* sp. nov. and *D. minor* sp. nov. by modified right and left legs 13, 15 or 17.

##### Description.

As for the genus, with the following details. Colour in alcohol uniformly pale yellow, sometimes with fine brown speckling at collum and paranotal margins. Adult male/female approximate measurements: length 9/8 mm, midbody diameter 0.9/0.8.

**Figure 13.**
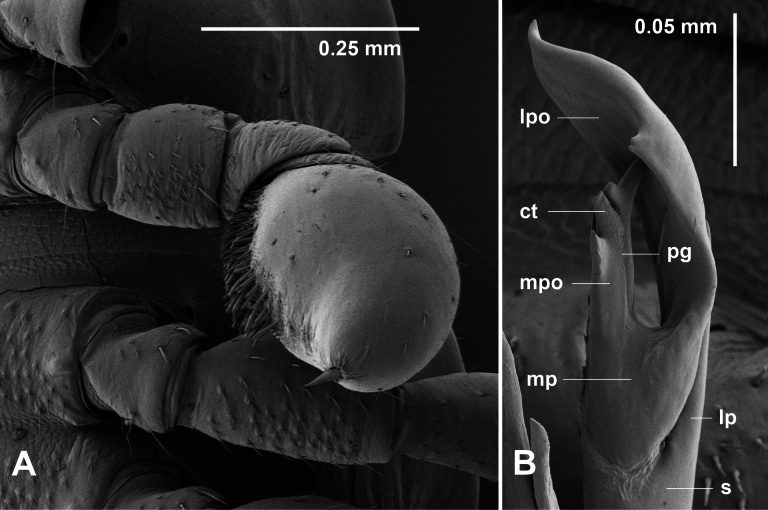
*Dibologonus oedipus* sp. nov., paratype male ex site HS40-C-L2, NMV K16526. **A.** Modified left leg 17; **B.** Posterior view of distal portion of left gonopod telopodite. Abbreviations: ct = central tooth, l = lateral process of solenomere, lpo = lateral portion of solenomere medial process, mp = medial process of solenomere, mpo = medial portion of solenomere medial process, pg = prostatic groove, s = solenomere.

Male with antenna almost reaching tergite 3 when manipulated dorsally; relative antennomere lengths 6 > 3 > 2 > (4,5); 6 widest. Posterior corners of paranota not projecting. Midbody metatergite width ca 1.2× prozonite width. Midbody leg ca 1.7× ring diameter. Legpairs 13, 15 or 17 may be greatly modified: prefemur, femur, postfemur and claw reduced in length, tibia and tarsus fused into swollen structure with dense ventral setation (Fig. 13A). Brush setae dense, tapering to slightly curved point, without mid-height notch, on coxa/trochanter, prefemur, femur; sphaerotrichomes with slightly flattened globular base and pointed shaft on postfemur, tibia and tarsus.

Gonopod telopodites (Figs 9D, 13B) straight, almost reaching legpair 5 when retracted. Telopodite divided at ca 1/3 telopodite height into medial branch and solenomere. Medial branch flattened, with small, tab-like posterolateral extension at ca 1/2 branch height; divided at ca 3/4 telopodite height into closely appressed medial and lateral processes: medial process shorter, flattened, tapering to blunt point; lateral process subcylindrical, tapering to blunt point a little distal to first division of solenomere. Solenomere well-separated from medial branch, divided at ca 7/8 telopodite height into medial and lateral processes, closely appressed, the lateral process tapering and terminating at level of prostatic groove opening on medial process. Medial process divided into leaf-like lateral portion curving medially, with small triangular extension on posterior margin, and well-separated, shorter, medial portion terminating in 3 small teeth, the central tooth having the prostatic groove opening and the most lateral tooth bluntly bifurcate.

Female a little smaller than male and with thinner, legs never modified; epigynum raised medially in low, rounded triangle; cyphopods not examined.

##### Name.

Noun in apposition, for the mythical king Oedipus of Thebes, whose name means “swollen foot”. See Remarks for notes on modified podomeres in this species.

##### Distribution.

Collected over a ca 125 km linear range from Namadgi National Park in the Australian Capital Territory to Kosciuszko National Park in New South Wales (Fig. 16A), in subalpine woodland and shrubland from 1485 to 1880 m.

##### Remarks.

Modified legpairs 13, 15 or 17 are found in males across the range of *D. oedipus* sp. nov. and are not restricted to particular areas. Among the males with modified legs, all specimens with intact legs have modifications on both the left and right posterior legs on a particular ring. Six males have modifications on leg 13 (ring 9), one on leg 15 (ring 10) and 17 on leg 17 (ring 11). A single male (with intact legs) has no leg modifications. Variation in which legs are modified appears within populations: site HS42-C-L1 had three males with modified leg 13 and one with modified leg 17, while site HS47-C-L2 had one male without modifications, one with modified leg 15 and two with modified leg 17.

Similarly swollen distal podomeres were reported by Shear et al. (2022) in two males of the chordeumatidan *Amplaria oedipus* Shear, Nosler and Marek, 2022 from Cape Mountain, Oregon, USA. In both specimens the swollen podomeres were on legpairs 5 and 6.

#### ﻿ Polydactylogonus gen. nov.

Taxon classificationAnimaliaPolydesmidaDalodesmidae

A506F4EB-E2B8-529E-BD1B-F635A515DBC2

https://zoobank.org/1544D3FE-FB19-49E0-BBC1-572229C40722

##### Type species.

*Polydactylogonus sanctogwinear* sp. nov., by present designation.

##### Other assigned species.

None.

##### Diagnosis.

Distinguished from other Australian dalodesmids by the slender gonopod telopodite ending in a fan-like cluster of five branches.

##### Description.

As for the type species.

##### Name.

Greek *polys* (many) + *dactylos* (finger), plus “-gonus”, an ending for genus names in Polydesmida that refers to gonopod structure, e.g. *Tridactylogonus* Jeekel, 1982. In anterior and posterior view the gonopod telopodite seems to end in five “fingers” spread apart. Masculine gender.

#### ﻿ Polydactylogonus sanctogwinear sp. nov.

Taxon classificationAnimaliaPolydesmidaDalodesmidae

E28D6FCB-8084-52B4-B0F7-C0D2DF01ECF2

https://zoobank.org/88234924-B8F3-439D-923A-B3405249B427

Figs 14, 15B

##### Type material.

***Holotype*.** Male, Baw Baw National Park (Vic), 0.26 km NNW of Mt St Gwinear carpark, site NP23-13, -37.8388, 146.3306 ± 25 m, 1325 m, coll. Nicholas Porch, 2023-03-15, 2 m^2^ litter sample from snow gum woodland, NMV K16527. ***Paratypes*.** 7M, details as for holotype, NMV K16531; 1M in 95% EtOH, details as for holotype, NMV K16528; 1M, same details but 0.14 km N by W of Mt St Gwinear carpark, site NP23-14, -37.8396, 146.3315 ± 25 m, 1315 m, NMV K16529; 4M, same details but 0.11 km W by N of Mt St Gwinear carpark, site NP23-15, -37.8407, 146.3307 ± 25 m, 1305 m, NMV K16530.

##### Additional material.

None.

##### Description.

**Figure 14.**
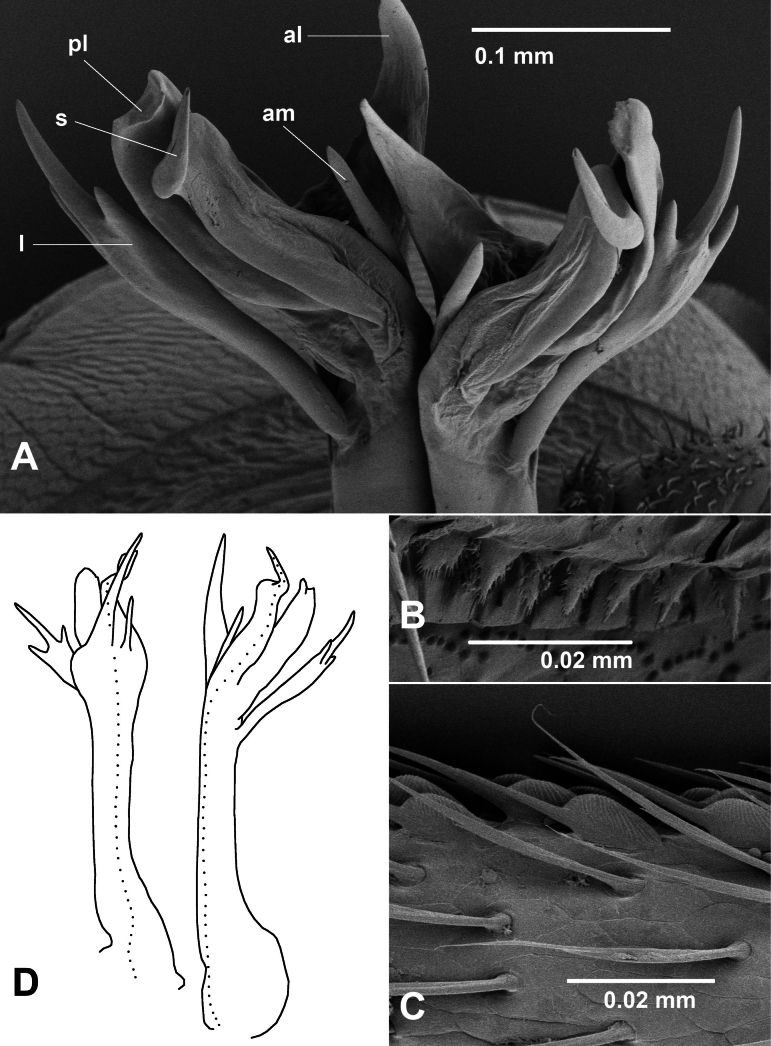
*Prodactylogonus sanctogwinear* gen. nov. et sp. nov.; paratype males ex site NP23-13, NMV K16531. **A.** Posterodistal view of gonopod telopodite tips in situ; **B.** Limbus on midbody ring; **C.** Sphaerotrichomes on leg 5 tarsus; **D.** Outline sketch anteromedial (left) and posterior (right) views of left gonopod telopodite; dotted line marks course of prostatic groove. Abbreviations: am = anteromedial branch, al = anterolateral branch, l = lateral branch, pl = posterolateral branch, s = solenomere.

Small dalodesmids ca 7 mm long with body plan head + 19 rings (including telson). Male colour in alcohol almost uniformly pale white, with reddish speckling at paranotal margins on some specimens. Head with vertex sparsely setose, frons and clypeus setose; vertigial sulcus reaching ca 1/2 way to level of line between top of antennal sockets. Sockets separated by ca 1.5× socket diameter. Antenna strongly clavate, reaching tergite 3 when manipulated. Relative antennomere lengths (6,3,2) > (4,5), 6 widest. Relative tergite widths 6 > 5 > 2 > 4 > 3 > collum; rings 7–16 subequal in width, 17 and 18 narrower. Collum much narrower than head, half-moon-shaped in dorsal view, corner rounded. Paranotal margin on ring 2 much lower than ring 3 margin; no pit ventrally under ring 2 paranotum. Midbody paranotal margin level, at ca 2/3 ring height; paranotum with anterior corner rounded, lateral margin gently convex with setae at three marginal notches, notches more obvious on posterior rings; posterior margin more or less straight; posterior corners not projecting. Midbody metatergite ca 0.6 mm wide, ca 1.2× prozonite width. Waist pronounced; prozonite posteriorly with small, tab-like elements clearly marking suture line; metatergites with three transverse rows of low bumps, the anterior bumps smallest, each bump bearing a single seta posteriorly; posterior margin of metatergite with 4^th^ transverse row of setae. Limbus composed of flat elements with minute, irregular, pointed branches (Fig. 14B). Pore formula normal; ozopore small, round, opening dorsally at posterolateral corner of paranotum. Epiproct extending a little beyond anal valves, conical, apex truncate. Spinnerets in square array, not in depression on epiproct. Hypoproct subtrapezoidal. Sternites slightly wider than long, transverse impression deeper than longitudinal impression. Spiracles small, round, very slightly raised above pleurite surface. Relative podomere lengths of midbody leg: tarsus >> femur >> prefemur > (postfemur, tibia); tarsus straight. Anterior legs somewhat swollen, prefemur and femur arched dorsally. Numerous sphaerotrichomes (Fig. 14C) on anterior leg tarsus, tibia; a few sphaerotrichomes on postfemur; sphaerotrichomes with flattened globular base, shaft straight, tapering, strongly declined. Brush setae dense on prefemur, femur tapering to blunt points, tips slightly curving. Gonopore opening distomedially on leg 2 coxa. Aperture ca 1/2 ring 7 prozonite width, anterior margin nearly straight, rim projecting laterally and posteriorly. Retracted gonopod telopodites reaching just past leg 6. Ring 6 sternite excavate with short setal brushes above leg bases supporting retracted telopodites.

Telopodites (Fig. 14A, D) erect, closely appressed, with a few basal setae posterolaterally. Telopodite divided at ca 2/3 height into fan-like cluster of 5 branches. Anteromedial branch short, spine-like. Anterolateral branch flat, long-triangular, directed slightly medially. Lateral branch cylindrical at base, expanding and flattening into fork-like group of 3 terminal processes tapering to points, the central process longest. Posterolateral branch flattened, with truncate tip bent towards solenomere. Posteromedial solenomere flattened, tapering abruptly and strongly near tip, the tip curving posteriorly, then distomedially. Prostatic groove running on medial surface of telopodite, following curve of solenomere to opening at apex.

##### Name.

Noun in apposition, for Mt St Gwinear.

##### Distribution.

So far known only from subalpine woodland from 1305 to 1325 m in a small area ca 2 km southeast of the summit of Mt St Gwinear, near Mt Baw Baw in the Victorian mountains (Fig. 16A).

##### Remarks.

No female specimens have been confidently assigned to this species.

### ﻿New locality records

#### ﻿ Cernethia inopinata

Taxon classificationAnimaliaPolydesmidaDalodesmidae

Mesibov, 2015

3EEBC1C2-0E41-52BA-9502-89CA8F068884

##### Material examined.

**Figure 15.**
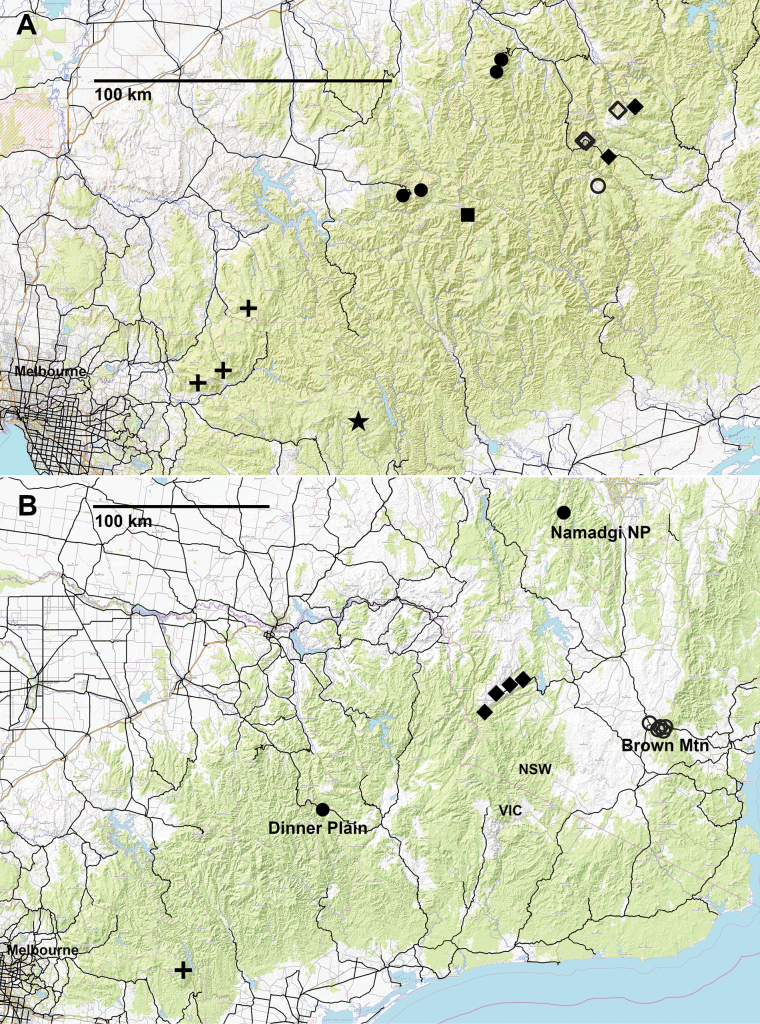
**A.** Localities for *Araneopedis bogong* sp. nov. (filled diamonds), *A. buffalo* sp. nov. (filled circles), *A. dargo* sp. nov. (open circle), *A. gibbae* sp. nov. (filled square), *A. porchi* sp. nov. (open diamonds) and *Lissodesmus milledgei* Mesibov, 2006: previously recorded (crosses) and new from this study (star); **B.** Localities for *Polydactylogonus sanctogwinear* sp. nov. (cross) *Cernethia dysmica* sp. nov. (diamonds) and *C. inopinata* Mesibov, 2015: previously recorded (open circles) and new from this study (filled circles).

3M, 3F, 45J from three sites in Namadgi National Park (ACT); 24M, 44F, 15J from a single site in the Dinner Plain area (VIC) (Fig. 15B). See Suppl. material 1 for details.

##### Remarks.

The type and previously known localities for *C. inopinata* (open circles in Fig. 15B) are in a cluster near Brown Mountain in eastern NSW. The new Namadgi National Park locality is ca 135 km to the northwest, but the new Dinner Plain locality in Victoria is more than 200 km southwest from the other localities. The Victorian records are also strange in that 83 *C. inopinata* specimens were collected in litter and pitfall samples at only one of the three HS08 sites (L/P3). At the other two HS08 sites, ca 250 m to the west, other millipede species were collected but not *C. inopinata*.

A likely explanation for these results is that *C. inopinata* has been accidentally introduced in the Dinner Plain area. Site HS08-O-L/P3 is close to a major tourist road (Great Alpine Road) and is ca 1 km west of Dinner Plain village, which has a cluster of holiday houses and a small hotel. It would be interesting to sample the Dinner Plain area in future to define the range limits of the local *C. inopinata* population and monitor any range expansion.

If *C. inopinata* was introduced via the Great Alpine Road, it may also have been introduced at the type and nearby localities, which are close to the Snowy Mountains Highway, and at the Namadgi National Park site, which is close to the Mt Franklin Road. The original range of this species is thus uncertain.

#### ﻿ Lissodesmus milledgei

Taxon classificationAnimaliaPolydesmidaDalodesmidae

Mesibov, 2006

B31CBFC0-EF47-53EB-8F76-B4130A3C0BC8

##### Material examined.

1M, 1F from two neighbouring sites on Mt Baw Baw (Vic) (Fig. 15A). See Suppl. material 1 for details.

##### Remarks.

The new records extend the range of this uncommon species ca 50 km to the southeast and upwards to ca 1430 m. It was previously known from three rainforest sites at elevations below 1000 m.

#### ﻿ Orthorhachis durabilis

Taxon classificationAnimaliaPolydesmidaDalodesmidae

Mesibov, 2008

8417A230-4B95-549D-948C-B2CAD727DDD8

##### Material examined.

**Figure 16.**
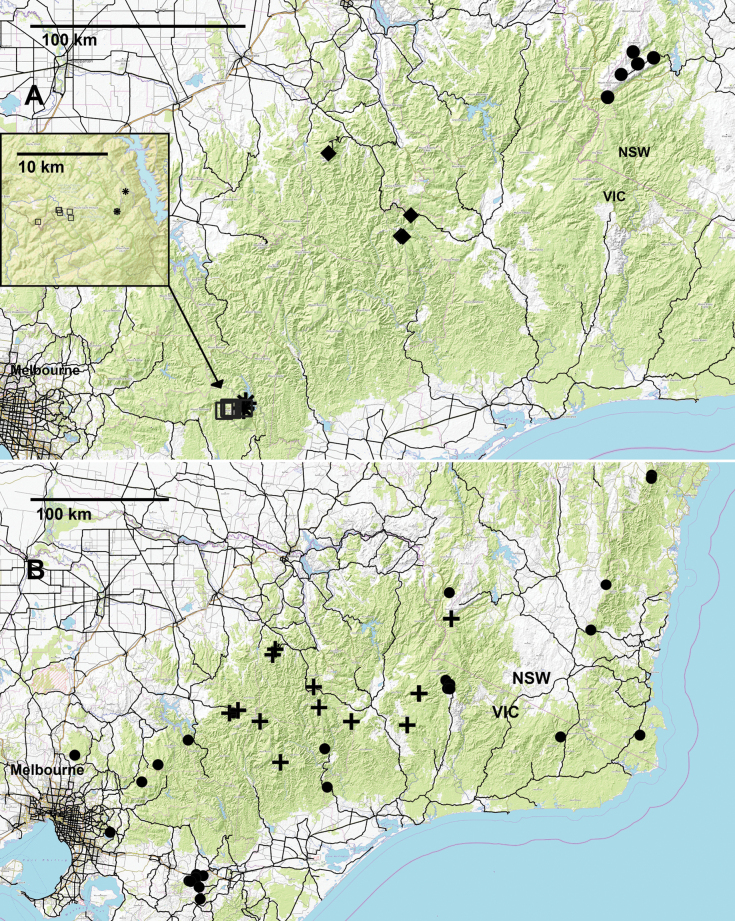
**A.** Localities for *Dibologonus major* sp. nov. (asterisks), *D. minor* sp. nov. (open squares), *D. oedipus* sp. nov. (circles) and *D. sladei* sp. nov. (diamonds). Inset shows localities for *D. major* sp. nov. and *D. minor* sp. nov.; **B.** Localities for *Orthorhachis durabilis* Mesibov, 2008: previously recorded (circles) and new from this study (crosses).

10M, 17F, 96J from 31 sites in the mountains of Victoria and New South Wales (Fig. 16B). See Suppl. material 1 for details.

##### Remarks.

The new records are in the mountainous parts of the *O. durabilis* range envelope and extend the known maximum elevation to ca 1760 m. This species is the most widespread dalodesmid on the southeastern Australian mainland, with a linear range extent of ca 480 km.

## ﻿Discussion and conclusions

The ten montane species in *Araneopedis* gen. nov., *Dibologonus* gen. nov. and *Polydactylogonus* gen. nov. were mainly collected by Tullgren funnel extraction from bulk litter samples. Extraction also yielded hundreds of specimens of juveniles and females that could not be assigned to species. Small, litter-dwelling dalodesmid species are remarkably similar, even in minor somatic details, and distinctive non-gonopodal characters are largely confined to males, such as presence/absence and structure of brush setae and sphaerotrichomes, and presence/absence of the ventral pit on tergite 2.

Whether any of the 11 species described here extend to lower elevations is currently not known. However, in Tasmania three alpine and subalpine dalodesmids have not been found at lower elevations: *Lissodesmus nivalis* Mesibov, 2018 (Mt Barrow, 1450–1550 m; Mesibov 2018), *L. piscator* Mesibov, 2019 (Ritters Plain, 1080 m; Mesibov 2019) and *Noteremus summus* Mesibov, 2009 (Mt Weld, 1100–1300 m; Mesibov 2009). If some of the new Victorian species are true high-elevation endemics, they may be at risk of extinction as the climate becomes warmer and drier in southeastern Australia.

## Supplementary Material

XML Treatment for Araneopedis

XML Treatment for Araneopedis porchi

XML Treatment for Araneopedis bogong

XML Treatment for Araneopedis buffalo

XML Treatment for Araneopedis dargo

XML Treatment for Araneopedis gibbae

XML Treatment for Cernethia

XML Treatment for Cernethia dysmica

XML Treatment for Dibologonus

XML Treatment for Dibologonus sladei

XML Treatment for Dibologonus major

XML Treatment for Dibologonus minor

XML Treatment for Dibologonus oedipus

XML Treatment for Polydactylogonus

XML Treatment for Polydactylogonus sanctogwinear

XML Treatment for Cernethia inopinata

XML Treatment for Lissodesmus milledgei

XML Treatment for Orthorhachis durabilis

## ﻿Additional information

### Use of AI

AI was not used.

### Author ORCIDs

Robert Mesibov https://orcid.org/0000-0003-3466-5038

### Data availability

All of the data that support the findings of this study are available in the main text or Supplementary Information.

## Supplementary materials

Supplementary material 1 Specimen records for named Dalodesmidae species from Hermon Slade Foundation project HSF23033 Data type tsv Explanation note The data table is a TSV in UTF-8 encoding and Darwin Core format. It contains 105 occurrence records for all new species of Dalodesmidae described in the accompanying article and in Mesibov (2025; https://doi.org/10.54102/ajt.gxasl), plus 38 new locality records for already described Dalodesmidae. The 47 Darwin Core fields are occurrenceID, type, basisOfRecord, datasetName, institutionCode, collectionCode, catalogNumber, kingdom, phylum, class, order, family, genus, specificEpithet, scientificName, taxonRank, identifiedBy, identifiedByID, dateIdentified, organismQuantity, organismQuantityType, organismRemarks, preparations, typeStatus, occurrenceStatus, establishmentMeans, eventID, continent, country, countryCode, stateProvince, locality, decimalLatitude, decimalLongitude, coordinateUncertaintyInMeters, geodeticDatum, coordinatePrecision, georeferenceSources, georeferencedBy, minimumElevationInMeters, maximumElevationInMeters, eventDate, recordedBy, recordedByID, locationRemarks, samplingProtocol and habitat. https://binary.pensoft.net/file/1489314This dataset is made available under the Open Database License (http://opendatacommons.org/licenses/odbl/1.0/). The Open Database License (ODbL) is a license agreement intended to allow users to freely share, modify, and use this Dataset while maintaining this same freedom for others, provided that the original source and author(s) are credited.Robert Mesibov
